# Induction of selective liver hypothermia prevents significant ischemia/reperfusion injury in Wistar rats after 24 hours ^1^

**DOI:** 10.1590/s0102-865020200020000005

**Published:** 2020-05-08

**Authors:** Tomaz de Jesus Maria Grezzana, Larisse Longo, Jorge Luiz dos Santos, Gemerson Gabiatti, Carlos Boffil, Emanuel Bendo dos Santos, Carlos Thadeu Schmidt Cerski, Marcio Fernandes Chedid, Carlos Otavio Corso

**Affiliations:** I PhD, Attending Liver Transplant Surgeon and Researcher, Liver Research Laboratory , Hospital de Clínicas de Porto Alegre (HCPA), Universidade Federal do Rio Grande do Sul (UFRGS), Porto Alegre - RS , Brazil . Design of the study, technical procedures, analysis of data, manuscript writing.; II PhD, Researcher, Liver Research Laboratory , HCPA , UFRGS , Porto Alegre - RS , Brazil . Conception and design of the study, technical procedures, manuscript writing.; III PhD, Pediatrics, Health Science Research Centre , University of Beira Interior (CICS, UBI), Covilhã , Portugal . Design of the study, analysis of data, manuscript writing.; IV PhD, Researcher, Liver Research Laboratory , HCPA , UFRGS , Porto Alegre - RS , Brazil . Design of the study, analysis of data, manuscript writing.; V MD, Researcher, Liver Research Laboratory , HCPA , UFRGS , Porto Alegre - RS , Brazil . Conception and design of the study, technical procedures, manuscript writing.; VI PhD, Attending, Kidney Transplant Surgeon and Researcher, Liver Research Laboratory , HCPA , UFRGS , Porto Alegre - RS , Brazil . Design of the study, analysis of data, manuscript writing.; VII PhD, Professor of Pathology, HCPA , UFRGS , Porto Alegre - RS , Brazil . Design of the study.; VIII PhD, Postgraduate Program in Surgical Sciences and Attending Liver Transplant Surgeon , HCPA , UFRGS , Porto Alegre - RS , Brazil . Design of the study, analysis of data, manuscript writing.; IX PhD, Postgraduate Program in Surgical Sciences, and Chair, Division of General Surgery , HCPA , UFRGS , Porto Alegre - RS, Brazil . Design of the study, technical procedures, analysis of data, manuscript writing. Abstract

**Keywords:** Cytokines, Hypothermia, Inflammation, Liver, Rats

## Abstract

**Purpose:**

To investigate the effects of induction of selective liver hypothermia in a rodent model.

**Methods:**

Seven male Wistar rats were subjected to 90 minutes of partial 70% liver ischemia and topic liver 26°C hypothermia (H group). Other seven male Wistar rats were subjected to 90 minutes of partial 70% normothermic liver ischemia (N group). Five additional rats underwent a midline incision and section of liver ligaments under normothermic conditions and without any liver ischemia (sham group). All animals were sacrificed 24-h after reperfusion, and livers were sampled for analyses. Pathology sections were scored for sinusoidal congestion, ballooning, hepatocelllular necrosis and the presence of neutrophilic infiltrates.

**Results:**

At the end of the experiment, liver tissue expressions of TNF-ɑ, IL-1β, iNOS and TNF-ɑ/IL-10 ratio were significantly reduced in the H group compared to N group, whereas IL-10 and eNOS were significantly increased in H group. Histopathological injury scores revealed a significant decrease in ischemia/reperfusion (I/R) injuries in H group.

**Conclusion:**

Selective liver hypothermia prevented I/R injury by inhibiting the release of inflammatory cytokines, preserves microcirculation, prevents hepatocellular necrosis and leukocyte infiltration, allowing maintenance of the liver architecture.

## Introduction

Ischemia/reperfusion (I/R) injury remains one of the major problems in liver surgery and transplantation ^1^ . In liver resections, the main cause of death after hepatectomy is postoperative liver failure. This may be secondary to a small liver remnant or to an impaired liver regeneration due to underlying liver disease ^2^ . Transient liver biochemical dysfunction always occurs after hepatic resection with or without pedicle clamping through Pringle maneuver. Ischemia induced by prolonged Pringle maneuver may also produce postoperative liver failure in extensive resections ^3^ .

In normal circumstances, liver tolerance to oxygen deprivation spans 45-60 minutes, depending on the presence of steatosis or chronic liver disease. However, this range may be extended to 120 minutes when liver hypothermia is induced ^3^ . The main technique for induction of hypothermia involves supra- and infrahepatic caval clamping, cannulation of portal vein trunk, infusion of chilled preservation solution and blood venting by a small orifice made either in the vena cava or suprahepatic veins. This technique is known as total vascular hepatic exclusion (TVHE) with *in situ* hypothermic perfusion. The organ is maintained cold during clamping, using ice slush over the liver surface. Body temperature is kept near-normal using external warming. Following these procedures, the resection is completed in a bloodless field. However, TVHE is technically and hemodynamically demanding, being restricted to major resections involving tumors near the confluence of hepatic veins and vena cava ^4^ . Thus, TVHE with *in situ* hypothermic perfusion is not widely utilized. Topical hypothermia (TH) is an easier alternative. In this technique, hypothermia is applied directly on liver surface during clamping of the liver pedicle ^5 - 6^ . In clinical practice, intermittent clamping of the liver pedicle is performed routinely during partial liver resection. Pringle maneuver is employed to prevent excessive bleeding from the raw surface of the liver. If proven as an effective practice, TH could serve as a tool to prevent hepatocellular damage.

Induction of hypothermia has the potential to reduce I/R injuries after organ reperfusion, potentially preventing liver failure in the postoperative period ^7^ . However, an incomplete understanding of the underlying protective mechanisms has limited a uniform and widespread implementation of liver-cooling techniques ^8^ . Mild hypothermia has consistently shown to provide protection against hepatic injury in different experimental models ^9 - 11^ . We hypothesize that induction of selective liver hypothermia would have protective effects on microcirculation and histopathological morphology, possibly by regulation of nitric oxide (NO) and nitric oxide synthase (NOS) pathways, and mediating inflammatory cytokines. This experimental study aimed to elucidate the potential protective mechanisms of TH in the ischemic liver 24 hours after reperfusion. The structural effects of TH in liver parenchyma 24 hours after reperfusion were also studied.

## Methods

All procedures were reviewed and approved by HCPA Ethics Committee, which follows the rules for animal experimentation advised by the Council for International Organization of Medical Sciences (CIOMS). This study is in accordance with the ARRIVE guidelines statement.

Male Wistar rats (n=19; weight 250-310 g) were raised and housed in the Experimental Animal Center at Hospital de Clínicas de Porto Alegre (HCPA), Brazil. Rats were housed at 22 ± 0.2°C, with a 12-h light/dark cycle and given food and water *ad libitum* . The rats were divided into 3 groups: sham (n=5); N, normothermic ischemia (n=7) and H, hypothermia 26°C (n=7). All animals were fasted 12 hours before the experiment, with free access to water.

### Experimental design and surgical procedures

The rats were anesthetized with a mixture of oxygen (0.5 l/min) and isoflurane (1-1.5%), and restrained supine on a warmed pad. A digital rectal thermometer was installed and body temperature was recorded every 15 minutes. A catheter was placed into the right carotid artery for measuring mean artery pressure (MAP) and for warmed saline bolus infusions to maintain MAP over 70 mmHg. In all groups, a midline incision was made and the liver ligaments were taken down. The anterior liver lobes were brought into a special device as previously demonstrated ^12^ and a wire thermometer was placed deep into the liver parenchyma. The vascular pedicle supplying the anterior lobes was clamped using an atraumatic clip for 90 minutes, but the vascularization to the posterior lobes remained intact to prevent intestinal congestion. In the H group, topical hypothermia was induced by dropping cooled saline on the surface of the anterior lobes. To prevent liquid spillage into the abdominal cavity and body cooling, appropriate aspirators were used, and body temperature was maintained at 36.5 ± 1°C in all animals, utilizing an auxiliary heating lamp and aluminum blankets as necessary.

After 90 minutes of ischemia, liver reperfusion was accomplished, and the hypotension caused by reperfusion syndrome was treated with saline infusions. The abdomen was closed and the animal recovered from anesthesia in a warm chamber. Twenty-four hours after reperfusion, the rats were anesthetized with isoflurane, the abdomen was opened, and the animals were sacrificed by exsanguination. Liver samples were obtained and were immediately frozen at -80°C for analyses.

### Assessment of inflammatory response

ELISA was used for assessing concentrations of TNF-α (Invitrogen – KRC3011), IL-1β (ThermoScientific – ER2IL1), IL-6 (Invitrogen - KRC0061) and IL-10 (Invitrogen - KRC0102). Liver tissue samples were homogenized in phosphate-buffered saline with added protease inhibitor (Sigma - SC-29131) in ice and centrifuged at 4000 rpm for 10 minutes at 4°C. The supernatant was collected for determining the concentration of the markers. All analyses were performed according to manufacturer’s directions. Absorbance was measured with a spectrophotometer at 450nm. Color intensity was directly proportional to cytokine concentration in the samples. Results were expressed in pg/mg protein.

### Assessment of microcirculatory injury

Cytoplasm extracts for determining eNOS and iNOS protein expressions by Western blot were prepared from liver tissue fragments using a protocol adapted from Sadowski & Gilman ^13^ . Briefly, liver samples were homogenized in ice with 500 µL of hypotonic buffer A (1 M HEPES, pH 7.9, 0.5 M NaF, 100 mM Na _3_ VO _4_ , 100 mM sodium glycerol phosphate, 0.5 M EDTA-Na, 100 mM EGTA and 1 mM DTT). Nonidet P-40 and protease inhibitor were added to this solution. Samples were centrifuged at 1000 rpm for 10 minutes at 4°C. The supernatant was obtained and stored at -80°C. Protein concentration in tissue homogenates was assessed according to the technique described by Bradford ^14^ . Absorbance was read with a spectrophotometer at 595 nm, and the values obtained were expressed in mg/mL.

### Western blot

Samples containing 100 μg of protein were separated by sodium dodecyl sulfate-polyacrylamide gel electrophoresis and transferred to polyvinylidene fluoride membranes. The membranes were then blocked with 5% nonfat dry milk in phosphate-buffered saline containing 0.05% Tween 20 (PBS-T) for 1 h at room temperature and probed overnight at 4°C with polyclonal anti-eNOS (SC8311/130kDa), monoclonal anti-iNOS (SC7271/ 120kDa) and polyclonal anti-β-actin (A5060/42kDa) antibodies at 1:200 - 1:1000 dilution with PBS-T in 5% nonfat dry milk. Afterwards, the membranes were washed with PBS-T and incubated for 90 minutes at room temperature with secondary HRP-conjugated antibody (Santa Cruz Biotechnology, Santa Cruz, CA, USA) at 1:5000 - 1:10,000 dilutions with PBS-T in 5% nonfat dry milk. Bands were visualized by chemiluminescence detection using a commercial ECL kit (WBKLS0050 Millipore Co., Billerica, MA, USA). The density of the specific bands was quantified with an L-Pix Chemi Molecular Imaging densitometer.

### Liver histology

Liver biopsies from anterior lobes were fixed in 4% buffered formalin, embedded in paraffin, and stained with hematoxylin-eosin (H&E). The specimens in each group were analyzed by an expert liver pathologist blinded to the study design. Sinusoidal congestion, ballooning, hepatocellular necrosis in zones 1 (centroacinar), 2 (intermediate) and 3 (periacinar), and neutrophilic infiltrate were assessed according to the degree of injury: 0 (no findings), 1 (minimal), 2 (mild), 3 (moderate) and 4 (severe), similar to the scale of Suzuki *et al* . ^15^ . The results are reported as the sum of the individual scores and separately for each parameter.

### Statistics

All data are expressed as means ± SEM. Data were analyzed using one-way ANOVA, and subsequently with the Holm-Sidak pairwise multiple comparison procedure. Differences were considered significant when *P* <0.05.

## Results

### Animal weight

Mean animal weight was 279.2 ± 21.3 g in the sham group, 291.6 ± 10.5 g in the N group and 272.6 ± 10.4 g in the H group (P=0.575).

### Liver weight

Livers were removed at the end of the experiment and immediately weighed. Mean liver weight in the sham, N and H groups at the end of the experiment was 9.85 ± 1.27, 11.43 ± 0.42 and 10.34 ± 0.28 g, respectively (P=0.177).

### Mean arterial pressure (MAP)

**Figure 1:**
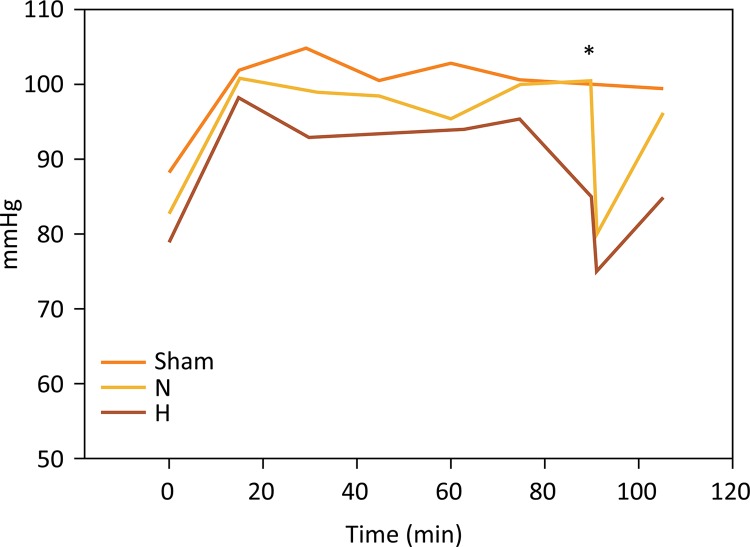
Mean arterial pressure (MAP) recordings (in mmHg) demonstrating no 570 significant effects of induction of liver hypothermia on hemodynamics during the procedure. 571 A slight decrease in MAP occurred after liver reperfusion*, followed by rapid recovery 572 (P=0.554; H, hypothermia at 26°C; N, normothermic ischemia. One-way ANOVA).

In all groups, MAP remained constant throughout the entire experiment. After reperfusion, there was a temporary decrease in MAP values, followed by fast recovery (Fig. 1). There was no significant difference between sham-operated controls (100 ± 5.6 mmHg), animals subjected to normothermic ischemia (89.7 ± 4.0 mmHg) and animals subjected to hypothermia (94.8 ± 8.3 mmHg) (P=0.554).

### Body temperature

**Figure 2:**
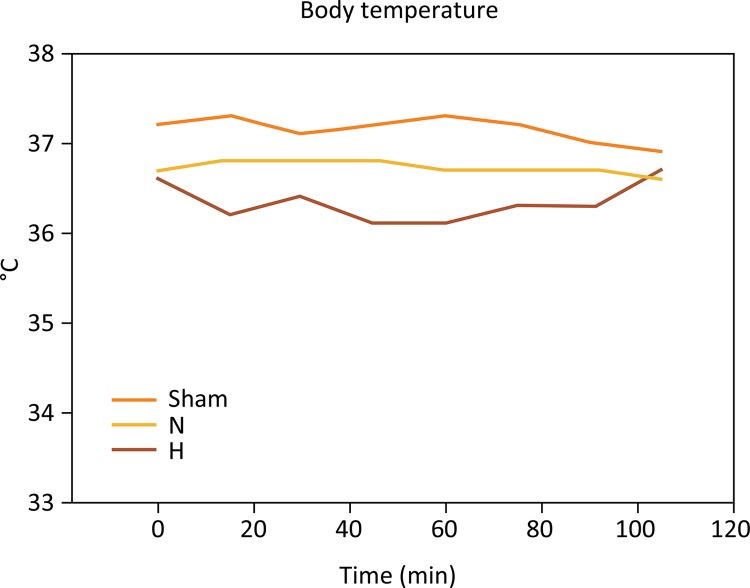
Body temperature remained stable during whole experiment. Despite the induction of selective liver hypothermia at 26°C, mean body temperature showed no differences among the groups (P=0.091). H, hypothermia at 26°C; N, Normothermic ischemia (One-way ANOVA).

Body temperature was continuously controlled during ischemia and kept within predetermined values (Fig. 2). There was no significant difference in the means of body temperature when the groups were compared: sham (37.1 ± 0.23°C), N (36.7 ± 0.16°C) and H (36.3 ± 0.3°C) (P=0.091).

#### Liver temperature

**Figure 3:**
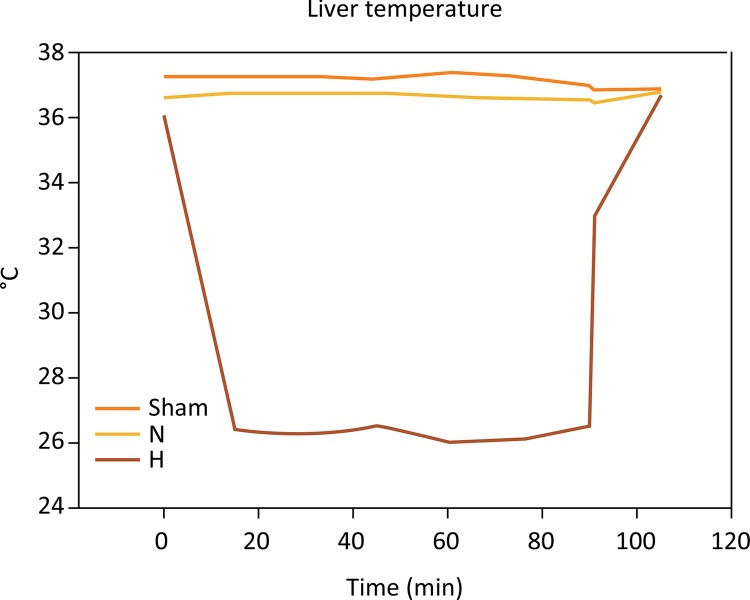
Liver temperature recordings during the experiment. A few minutes were necessary to reach the target of 26°C. The H group demonstrated a significant difference in comparison to shamand normothermic groups (P<0.001 and P<0.001, respectively). H, hypothermia at 26°C; N, Normothermic ischemia (One-way ANOVA).

Recorded each 15 min, mean liver temperature (Fig. 3) in H group was significantly lower as compared to N and sham controls (29.3 ± 0.32 *vs* . 37.1 ± 0.28 and 36.7 ± 0.18°C, P<0.001 and P<0.001, respectively).

## Liver cytokines

**Figure 4:**
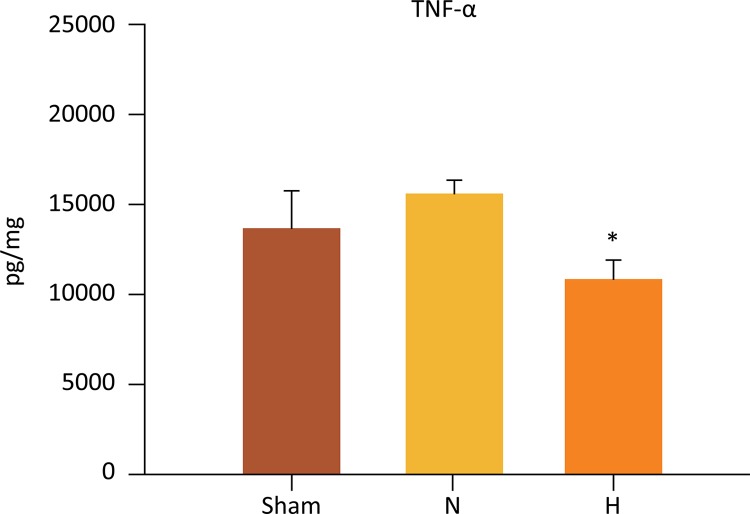
Selective liver hypothermia at 26°C inhibited the release of the pro-inflammatory mediator TNF-α (Values are expressed as the mean ± SEM *P=0.044, H *vs* . N group). TNF-α; tumor necrosis factor alpha.

The cytokine expression levels in the liver homogenates at the end of the experiment are shown in Figure 4 . TNF-α expression in the liver was significantly lower in the H group as compared to the N group (10,900 ± 1013 *vs* . 15,589 ± 823 pg/mg, respectively, P=0.044). When H and N groups were compared to sham, no differences were observed (10,900 ± 1013 and 15,589 ± 823 *vs* . 13,590 ± 2167 pg/mg, =0.315 and P=0.304, respectively).

**Figure 5:**
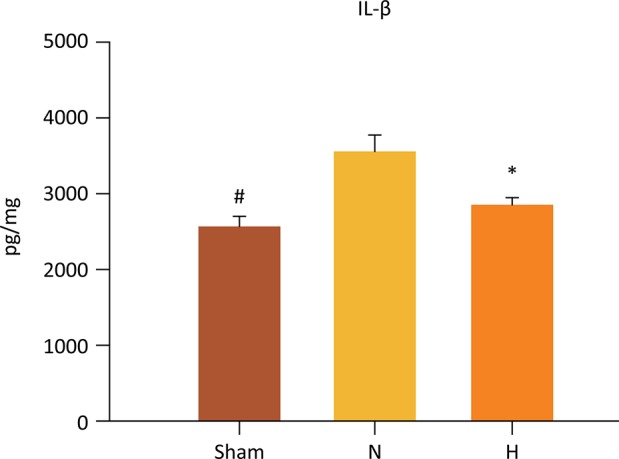
Selective liver hypothermia at 26°C inhibited the release of the pro-inflammatory mediator IL-1β in liver I/R injury (Values are expressed as the mean ± SEM). #P=0.002, Sham *vs* . N; *P=0.009, H *vs* . N group; IL-1β, interleukin 1 beta, H, hypothermia 26°C; N, normothermic ischemia (One -way ANOVA).

Accordingly, IL-1β levels in the liver were significantly lower in the H (2833±91 pg/mg) and sham groups (2551±133 pg/mg ) as compared to N group (3538 ± 211 pg/mg), (P=0.009 and P=0.002, respectively) (Fig. 5). When the H and sham groups were compared, no significant differences were observed (2833 ± 91 *vs* . 2551 ± 133.2 pg/mg, respectively, P=0.247).

**Figure 6:**
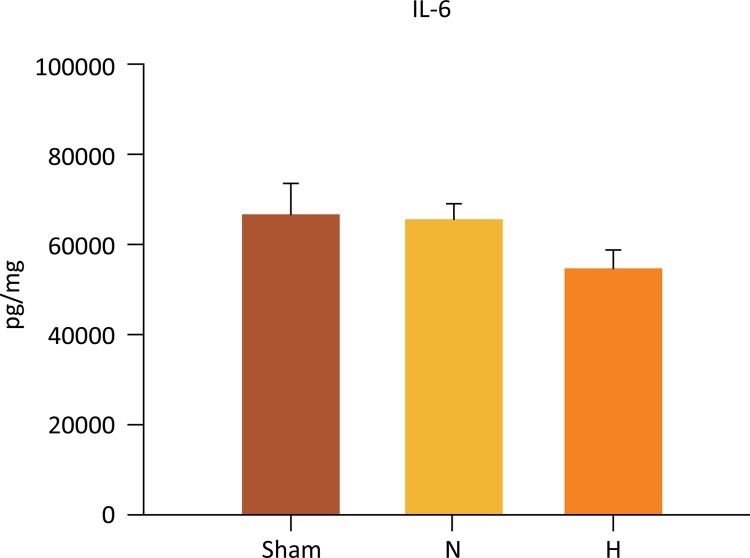
Selective liver hypothermia at 26°C had no effect in liver IL-6 expression (P=0.293).

IL-6 levels in liver tissue were similar in all groups (sham: 68 ± 7 *vs* . N: 67 ± 3.5 *vs* . H: 56 ± 4 pg/mg, respectively, P=0.293) (Fig. 6).

**Figure 7:**
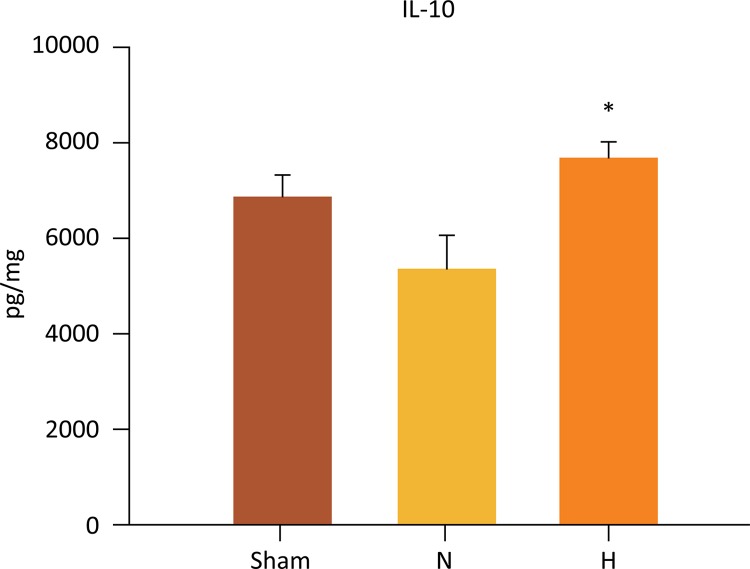
Selective liver hypothermia at 26°C preserved anti-inflammatory IL-10 expression in liver I/R injury (Values are expressed as the mean ± SEM). *P= 0.020, H *vs* . N group; IL-10, Interleukin 1.

IL-10 levels in the liver were significantly higher in the H group as compared to N group (7672± 349 *vs* . 5357 ± 705 pg/mg, P=0.020) (Fig. 7). No differences were observed when the H group was compared to sham group (7672 ± 349 *vs.* 6842 ± 500 pg/mg, P=0.324). Also, no difference was found when N group was compared to sham group (5357 ± 705 *vs* . 6842 ± 500 pg/mg, P=0.167).

## TNF-α/IL-10 ratio

**Figure 8:**
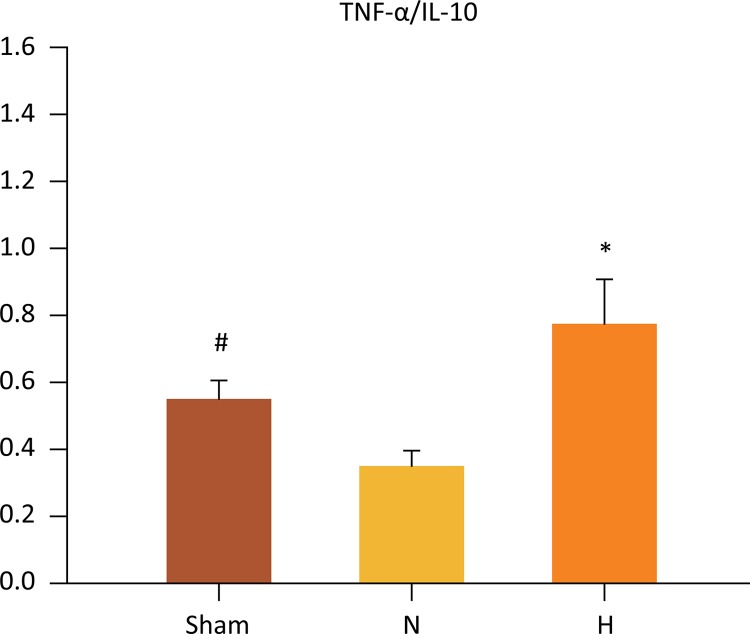
Selective liverhypothermia at 26°C promoted a favorable balance between pro- and anti-inflammatory cytokines, as expressed by the (E) TNF-α/IL- 10 ratio. Values are expressed as the mean ± SEM. # P=0.024, Sham *vs* . N; *P=0.002, H *vs* . N group (One-way ANOVA). H, hypothermia 26°C; N, normothermic ischemia; TNF-α; tumor necrosis factor alpha; IL-10, Interleukin 1.

As a reflection of pro- and anti-inflammatory balance, the TNF-α/IL-10 ratio (Fig. 8) was significantly higher in H group as compared to N group (1.46 ± 0.17 *vs.* 3.20 ± 0.41, P=0.002). I also was lower in the sham group as compared to N group (and 1.95 ± 0.21 *vs* . 3.20 ± 0.41, P=0.024). No significant differences were observed when H and Sham groups were compared (1.46 ± 0.17 *vs* . 1.95 ± 0.20, P=0.281).

## Liver eNOs, iNOS and eNOS/iNOS ratio

**Figure 9:**
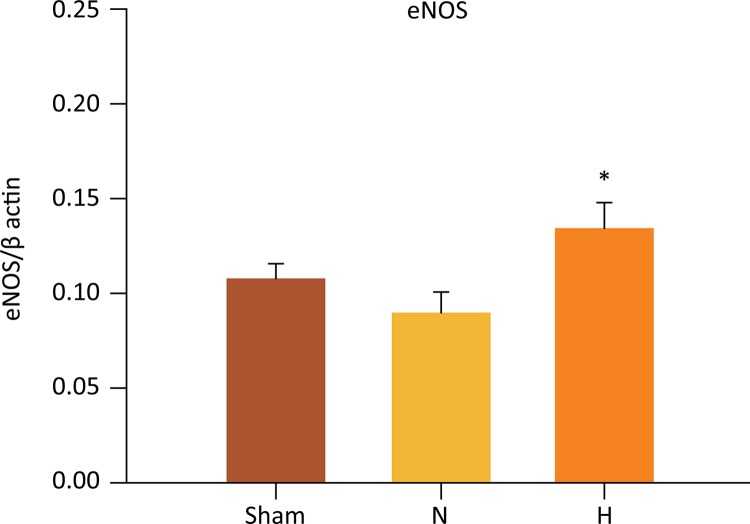
Selective liver hypothermia at 26°C regulates the activity of eNOS. (Values are expressed as the mean ± SEM). *P=0.039, H *vs* . N group (One -way ANOVA). H, hypothermia 26°C; N, normothermic ischemia; eNOS, Endothelial Nitric Oxide Synthase.

Hepatic levels of eNOS were significantly higher in the H group as compared to N group (0.134 ± 0.01 *vs* . 0.089 ± 0.01 eNOS, P=0.039) (Fig. 9). There were no significant differences in eNOS levels between the H and sham groups (0.134 ± 0.01 *vs.* 0.108 ± 0.008, P=0.321). Accordingly, there were no differences between N and sham groups (0.089 ± 0.01 *vs* . 0.108 ± 0.008, P=0.346).

**Figure 10:**
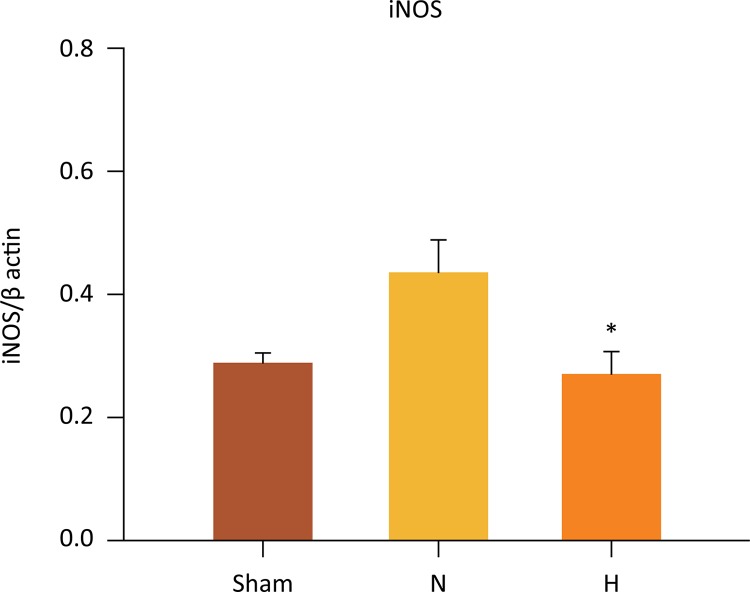
Selective liver hypothermia at 26°C regulates the activity iNOS. (Values are expressed as the mean ± SEM). P =0.040, H *vs* . N group; H, hypothermia 26°C; N, normothermic ischemia; iNOS, Inducible Nitric Oxide Synthase (One-way ANOVA).

In contrast, hepatic iNOS protein expressions were significantly lower in the H group as compared to N group (0.267 ± 0.04 *vs* . 0.435 ± 0.05 iNOS, P=0.040) (Fig. 10). No significant differences in iNOS were observed between the H and sham groups (0.267 ± 0.04 *vs.* 0.288 ± 0.01, *p* =0.763). Accordingly, there no differences between N and sham groups (0.435 ± 0.05 *vs* . 0.288 ± 0.01, P=0.108).

**Figure 11:**
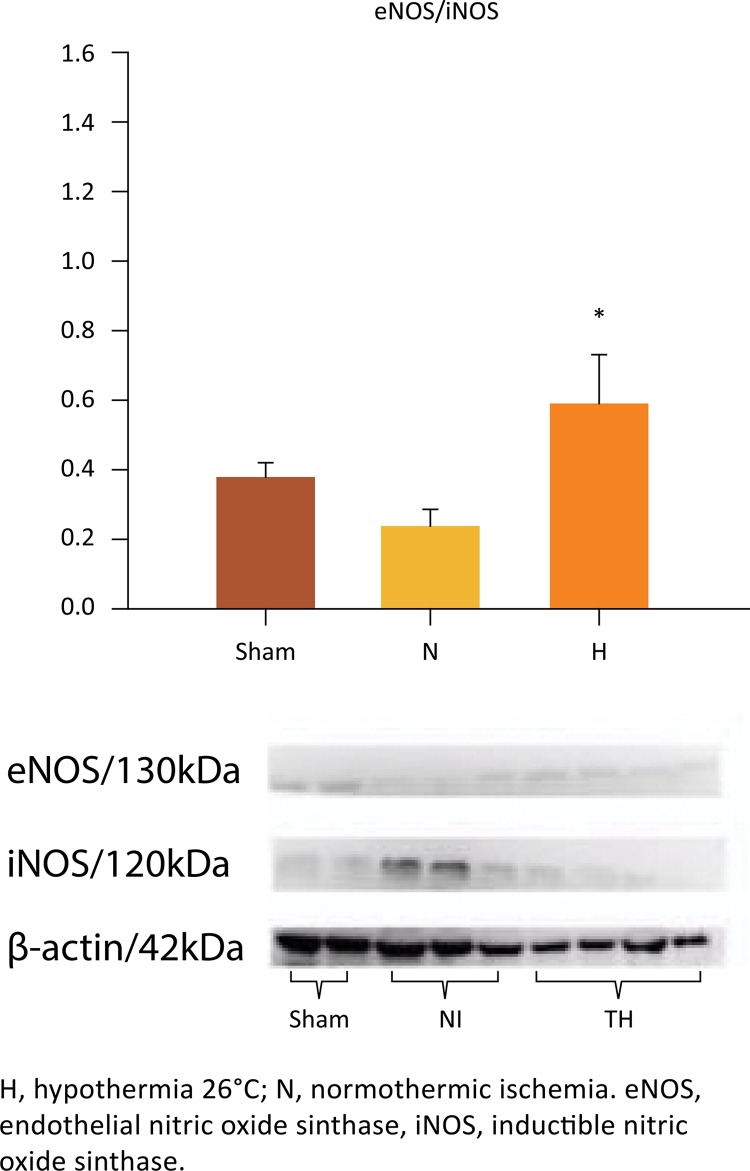
- Selective liver hypothermia at 26°C promotes a favorable balance in microcirculation expressed as eNOS/iNOS ratio. (Values are expressed as the mean ± SEM). *P<0.024 *vs* . N group (One-way ANOVA).

The eNOS/iNOS ratio in the H group was significantly higher as compared to N group (0.405 ± 0.14 *vs* . 0.245 ± 0.05, P=0.024), but no differences were observed when the H and sham groups were compared (0.405 ± 0.14 *vs* . 0.380 ± 0.05, P=1.0) (Fig. 11). There were no differences between N and sham groups (0.245 ± 0.05 *vs.* 0.380 ± 0.05, P=0.321). The results are displayed in Figure 11 .

## Histopathological Injury scores

**Figure 12:**
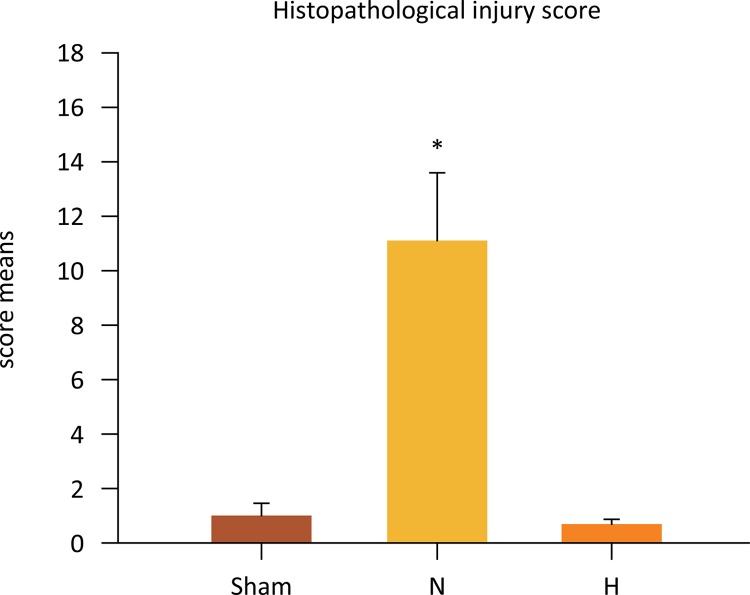
Selective liver hypothermia at 26°C reduces liver histopathological injury score (Values are expressed as the mean ± SEM). #P=0.001, N *vs* . Sham; *P<0.001, N *vs* . H group (One -way ANOVA). H, hypothermia 26°C; N, normothermic ischemia.

The sum of the means for all parameters evaluated was converted into a score. This score is shown in Figure 12 . There was a significant difference when the H and N groups were compared (0.71 ± 0.18 *vs.* 11.14 ± 2.44, P<0.001) and when the N and Sham groups were compared (11.14 ± 2.44 *vs* . 1.0 ± 0.44, P=0.001). No differences were observed between H and sham (0.71 ± 0.18 *vs.* 1.0 ± 0.44, P=0.904).

**Figure 13:**
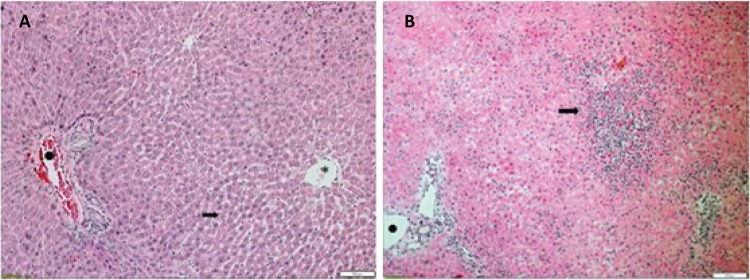
A) Liver subjected to ischemia under hypothermia 26°C depicting a preserved structural architecture ( *black arrow*: small area of sinusoidal congestion; black cross: portal space; *asterisk*: sinusoidal vein). B) Liver subjected to normothermic ischemia showing extensive coagulation necrosis and damaged parenchymal architecture ( *black arrow*: neutrophilic infiltrate surrounded by extensive areas of hepatocellular necrosis; *black cross*: portal space) H&E, x10.

Each selected parameter of I/R injury also was analyzed separately. The injury grade of each parameter is depicted in Fig. 13. No significant differences were observed among the groups in sinusoidal congestion and ballooning (P=0.08, P=0.781, respectively). Hepatocellular necrosis in zone 1 was significantly higher than those of the other two study groups (P=0.045). However, multiple pairwise comparison revealed a trend toward a difference (N *vs.* H, P=0.077; N *vs.* sham, P=0.078; and H *vs.* sham, P=1.0). Hepatocellular necrosis in zone 2 was significantly higher in the N group as compared to H and sham groups (2.86 ± 0.74 *vs.* 0.00 ± 0.00 and 0.00 ± 0.00, P=0.001 and P=0.002, respectively). No differences were observed when the H and Sham groups were compared (P=1.0). Accordingly, hepatocellular necrosis in zone 3 was significantly greater in the N group as compared to H and Sham groups (2.86 ± 0.74 *vs.* 0.00 ± 0.00 and 0.00 ± 0.00, P<0.001 and P=0.001, respectively). Furthermore, neutrophilic infiltrate showed a significantly higher score in the N group as compared to H and sham groups (2.58 ± 0.58 *vs.* 0.00 ± 0.00 and 0.00 ± 0.00, P<0.001 and P<0.001, respectively).

## Discussion

In the present study, the effects of selective liver hypothermia at 24 hour after reperfusion were investigated. The present study is a continuation of our previous work, which demonstrated that selective liver hypothermia applied by topical cooling offers protection against I/R injuries in a 2-hour reperfusion model ^12^ . This study was the first one assessing these injury scores after 24 hours under TH.

The ability of hypothermia at decreasing tissue damage was evaluated in mice 8 hours after hypothermia by Kato *et al* . ^26^ . The authors showed that hypothermia reduced neutrophilic infiltrates in up to 99% in liver sections. Similarly, Heijnen *et al* . ^16^ showed a decrease in scores of injuries in pigs employing an *in situ* hypothermia experimental model. Our findings corroborate the high capacity of TH of blocking innate inflammatory response. Overall, liver architecture was maintained intact after 24 hours in the hypothermic group, markedly in contrast to the normothermic group.

The experimental model utilized in this study is associated with substantial hepatocellular necrosis ^16^ . Notably, 26°C selective liver hypothermia practically abolished hepatocellular necrosis, in contrast to the normothermic group. Given the extensive body of evidence on the evaluation of this subject, it is conceivable that ATP preservation may be the responsible mechanism. Several studies have shown that when hypothermia is induced in the liver under ischemia, even a mild decrease in core liver temperature is able to improve hepatocellular function, reduce histopathological damage, decrease systemic inflammatory response, attenuate oxidative stress, preserve microcirculation integrity and increase survival ^9 , 15 - 21^ . It has been further demonstrated that the protective effects of hypothermia are optimal when the ischemic liver is cooled to 26°C, whereas lower temperatures do not add hepatoprotection ^9 , 20^ . There is evidence that hypothermia positively influences the energy status of hepatocytes under ischemia, preserving ATP and avoiding the deleterious effects of the I/R phenomenon ^19^ . Our observations suggest that the induction of selective liver hypothermia acts mainly in the ischemic phase ^22^ and such protection prevents the injuries usually observed in the late phase of reperfusion. Considering that ATP is essential for liver recovery after a ischemic insult, the maintenance of ATP stores during ischemia permits fast recovery in reperfusion ^23^ and prevention of hepatocellular necrosis, release of damage-associated molecular patterns (DAMPs) into the circulation and triggering of the innate immune system ^8^ . Reports have corroborated these hypotheses and the attenuation of hepatocellular necrosis has been demonstrated under hypothermic conditions ^24^ . Accumulating experimental evidence demonstrates that liver hypothermia has a critical role in protecting mitochondrial bioenergetics and conservation of mitochondrial membrane permeability transition (MPT) ^22^ . In hepatocytes stressed by ischemia, ATP depletion and overproduction of reactive oxygen species (ROS) together decrease the threshold for MPT and lead to hepatocellular necrosis ^25^ .

Bile acid canalicular transport is similarly affected by I/R ^22^ . The findings of the present study are consistent with our previous data, which demonstrated attenuation of oxidative stress and recovery of bile flow 2 hours after reperfusion, a process involving biliary canalicular transport, which is highly dependent on ATP ^12^ . Our study found that the protective effects of hypothermia were even more marked in lobular zones 2 and 3. Similar findings were reported by Kato *et al.* ^26^ , who found patchy areas of necrosis in zones 2 and 3 and massive disruption of lobular morphology in conditions of normothermic ischemia, effects suppressed by hypothermia. In contrast, there were no significant differences in hepatocellular damage in lobular zone 1. The possible explanation is that this area is the nearest one of portal triads, which receives a higher oxygen supply, as previously demonstrated ^8^ .

After reperfusion, TNF-α and IL-1β released by activated Kupffer cells (KC) are of particular importance to start and propagate the inflammatory response, leukocyte rolling and adhesion to the endothelium ^27^ . A significant decrease in the expression of these cytokines in the hypothermic group in our study suggests that liver cooling attenuated the activation of KC at initial reperfusion and suppressed its subsequent deleterious effects.

TNF-α induces expressions of adhesion molecules on endothelial cells, stimulates chemokines such as CXC and thus the recruitment of neutrophils, which leads to further increased production of ROS and proteases, promoting additional injury ^8^ . In addition to the reduced expression of pro-inflammatory cytokines, the present study revealed significant differences in the expression of IL-10 among the groups. IL-10 is a potent anti-inflammatory cytokine, which regulates the release of other pro-inflammatory chemokines by various cell types ^28^ . In endotoxemic rats, an increased survival is related to augmented plasma IL-10 expression ^29^ . Furthermore, IL-10 pretreatment in obese rats limits the production IL-1β and increases survival after ischemia ^28^ . Interestingly, IL-10 is itself an inducer of hypothermia and its antagonism leads to augmented expressions of TNF-α, IL-1β and IL-6 ^30^ . Although KC are associated with I/R injuries, these cells may maintain a homeostatic level of inflammation by production and modulation of IL-10, suppressing endothelial activation. IL-10 production is a key factor to the regulation of leukocyte-endothelial cell interaction after various insults, in particular those mediated through toll-like receptors (TLR 4) and endotoxemia ^28^ . Our data are in line with those studies and show that liver cooling modulates the expression of IL-10 in this organ and blocks the occurrence of inflammation in reperfusion. In addition, we measured for the first time the TNF-α/IL-10 ratio in liver under hypothermic conditions.

TNF-α/IL-10 has been considered a surrogate marker of immune homeostasis in clinical situations, such as myocardial infarction and hyperglycemic states in pregnancy and may predict the occurrence of infections in burn injuries ^31^ . It is conceivable that the balance of these cytokines may be useful in states of I/R injury, allowing the equalization of intra- and inter-individual variability with respect to pro- and anti-inflammatory activities ^31^ . Our data demonstrate that selective liver hypothermia acts as a potent anti-inflammatory tool, and that TNF-α/IL-10 ratio may be a useful biomarker of neutrophilic infiltration and possibly liver dysfunction after prolonged pedicle clamping.

IL-6 was extensively studied in models of initial reperfusion, but very few studies have shown its expression in late reperfusion. Liver IL-6 remained unchanged after 24 hours after reperfusion in both groups. There are some conflicting data about the role of IL-6 in the context of hypothermia. In experimental models, moderate hypothermia was associated with decreased levels of IL-6 and hepatoprotection, but mild hypothermia showed no differences in models of hemorrhagic shock ^32^ . Our findings are similar to those of Horst and coworkers, who assessed IL-6 levels in the liver and plasma after induced trauma after 14, 24 and 48 hours under normothermic and hypothermic conditions in pigs, and no significant differences were observed in systemic IL-6 concentrations ^33^ . The explanation for the discrepancies in systemic or local expressions of IL-6 is possibly related to differences in diverse experimental models and times of observation.

Basally, eNOS induces vasodilation at the level of the pre-sinusoids and sinusoids ^34^ and also prevents platelet adhesion, thrombosis and polymorphonuclear leukocyte accumulation ^35 - 38^ . Our data showed that selective liver hypothermia plays a critical role in the regulation of the liver microcirculation, which involves NOS ^39^ . Various studies have demonstrated the crucial role of NOS in attenuating I/R injuries, by microcirculation regulation, reduction of macrophage infiltration, protection of hepatocytes from apoptosis and inhibition of the pro-inflammatory cascade ^40 - 44^ . Experimental evidence indicates that nitric oxide generated by the eNOS isoform protects against liver I/R injury. In eNOS knockout mice, a significantly greater liver injury was observed following warm I/R when compared to wild-type animals ^42^ . On the other hand, mice with transgenic overexpression of eNOS showed greatly reduced liver I/R injury compared to wild-type animals ^44^ . The protective effects of eNOS overexpression raise the possibility that I/R injury may act by down-regulating the expression of eNOS. Consistent with previous studies, we observed that the expression of eNOS was upregulated when liver hypothermia was induced under ischemia.

The role of iNOS in liver I/R injury is much less clear than that of eNOS ^41 , 45 - 48^ . There is some evidence that the effects of iNOS expression on liver I/R injury might be dependent on the duration of liver ischemia. In the present study, down-regulation of iNOS after 90 minutes of TH was detected after 24 hours of reperfusion. In contrast, normothermic ischemia caused a significant increase in liver expression of iNOS. This finding entails that under normothermic ischemia, the microcirculation damage will be sustained for even a period longer than 24 hours, and selective liver hypothermia is able to abrogate such response, thus preventing injury. The mechanisms underlying the deleterious effects of iNOS during prolonged ischemic insults are linked to excessive iNOS-derived NO generation in KC and neutrophils ^49^ and consequent promotion of pro-inflammatory cascade ^50^ . Specifically, the excessive generation of NO that occurs after ischemia promotes and increases release of radical superoxide (O _2_ •-) ^28^ , resulting in formation of peroxynitrite (ONOO ^-^ ), a molecule that is extremely toxic to cells ^51^ .

There are scarce reports evaluating the role of eNOS/iNOS ratio in endothelial dysfunction under hypothermic conditions. In the current study, TH promoted a significant increase in the eNOS/iNOS ratio. This finding was associated with preservation of both the histology and the function of the microcirculation, even after 24 hours after reperfusion. In addition to protection of the microcirculation, TH seems to play a decisive role in the balance of nitrosative stress ^18 , 52^ .

There are concerns that body hypothermia could trigger deleterious clinical effects, such as coagulopathy and susceptibility to infection. However, evidence in acute liver failure argues against these complications ^53^ . Studies investigating the effect of selective cooling of the liver in the setting of acute liver failure are under way in our laboratory. In addition, TH has the advantages of being fast, easily reproducible and of low-cost. Utilization of TH could yield higher benefits in liver resections involving patients suffering from chronic liver diseases, such as severe steatosis and cirrhosis ^24^ .

## Conclusions

In this experimental model using prolonged clamping, liver injury was significantly attenuated by induction of selective liver hypothermia. The potential protective mechanisms involved are likely related to preservation of hepatic microcirculation, inhibition of inflammatory response, and maintenance of the liver architecture. In clinical practice, TH could be employed routinely as an adjunct to Pringle maneuver during partial liver resections.
