# Erratum

**DOI:** 10.1590/s0102-865020200020000005err

**Published:** 2020-06-02

**Manuscript:** Induction of selective liver hypothermia prevents significant ischemia/reperfusion injury in Wistar rats after 24 hours

**Publication:** Acta Cir Bras. 2020;35(2):e202000205

**Doi:** http://dx.doi.org/10.1590/s0102-865020200020000005

On the first page of the original publication, instead of this authors names:

Tomaz de Jesus Maria Grezzana-Filho^I^, Larisse Longo^II^, Jorge Luiz dos Santos^III^, Gemerson Gabiatti^IV^, Carlos Boffi^lV^, Emanuel Bendo dos Santos^VI^, Carlos Thadeu Schmidt Cerski^VII^, Marcio Fernandes Chedid^VIII^, Carlos Otavio Corso^IX^

Consider this names:

Tomaz de Jesus Maria Grezzana-Filho^I^, Larisse Longo^II^, Jorge Luiz dos Santos^III^, Gemerson Gabiatti^IV^, Carlos Boffi^lV^, Emanuel Burck dos Santos^VI^, Carlos Thadeu Schmidt Cerski^VII^, Marcio Fernandes Chedid^VIII^, Carlos Otavio Corso^IX^

